# Stage IV Small Lymphocytic Lymphoma Presenting with Unilateral Vision Loss

**DOI:** 10.1155/2020/3752409

**Published:** 2020-01-11

**Authors:** Christopher Le, Adam Jacob, Devarajan Iyengar, John Dedousis, Antonios Tsompanidis

**Affiliations:** Department of Graduate Medical Education, CarePoint Health Bayonne Medical Center, 29 E. 29 Street, Bayonne, NJ 07002, USA

## Abstract

Small lymphocytic lymphoma (SLL) is a manifestation of chronic lymphocytic leukemia (CLL) in which malignant B-cell lymphocytes accumulate in the lymph nodes or bone marrow. In this report, we describe the medical course of a patient diagnosed with stage IV small cell lymphocytic lymphoma, who presented to the emergency room with acute neurologic manifestations of SLL.

## 1. Introduction

SLL is a form of non-Hodgkin lymphoma that involves malignant proliferation of B-cells in lymph nodes and other lymphoid tissues. SLL is often categorized along with CLL because they share many of the same clinical features but differ in the site of B-cell proliferation. CLL/SLL is the most common form of leukemia in the United States, making up about 25-30% of all diagnosed leukemia cases [1]. These leukemia cases have a stronger disposition for males compared to female, with the ratio of cases about 1.5 : 1 [1], most often diagnosed in the elderly population > 60 years old. The incidence of SLL is very low in patient categorized with CLL, as about 10% of patient have only nodal involvement.

Most patients present asymptomatically, with leukocytosis found incidentally on routine blood analysis. The patient may show symptoms of anemia, thrombocytopenia, chronic infections, hepatosplenomegaly, or lymphadenopathy at presentation, but these are much less common than the asymptomatic patient. More rarely, patients present with classic B-symptoms including fever, unexplained weight loss, or night sweats. Because SLL is an uncommon, indolent disease, patients are often asymptomatic for years and may eventually present due to complications of metastases.

## 2. Case Presentation

**Figure 1:**
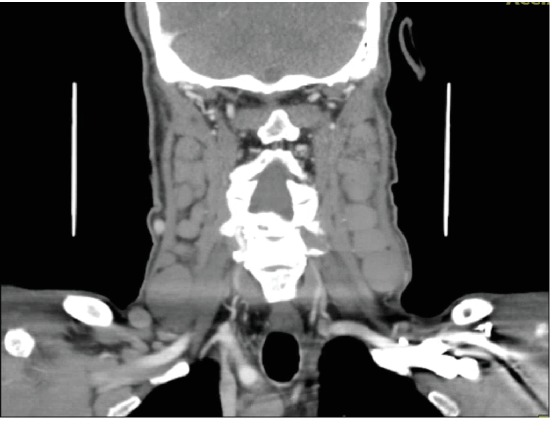
CT of the soft tissue in the neck showing diffuse cervical lymphadenopathy.

The patient is a 71-year-old Hispanic male with a past medical history of hypertension, COPD, and prostate cancer in remission post-radiation presented to the emergency department in May 2017 with a one-month history of neck swelling, sore throat, and ear pain. CT scan of the neck soft tissue showed multiple enlarged cervical lymph nodes in the submandibular, submental, posterior cervical, and jugulodigastric regions extending into the supraclavicular area (Figure 1). On the exam, the patient had left posterolateral oral pharyngeal soft tissue enlargement and possible left palatine tonsil involvement. Unfortunately, he left against medical advice before any further evaluation could be performed.

In November 2017, the patient returned to the emergency department with a 2-day history of headache, neck pain, painless vision loss in the right eye, and enlarging cervical and inguinal lymphadenopathy. Significant findings on examinations were complete right eye blindness and bilateral nontender lymphadenopathy in the cervical, supraclavicular, and axillary regions. On fundoscopic examination of the right eye, no retinal hemorrhages were appreciated. However, there was concern for optic nerve swelling and possible increased intraocular pressure.

**Figure 2:**
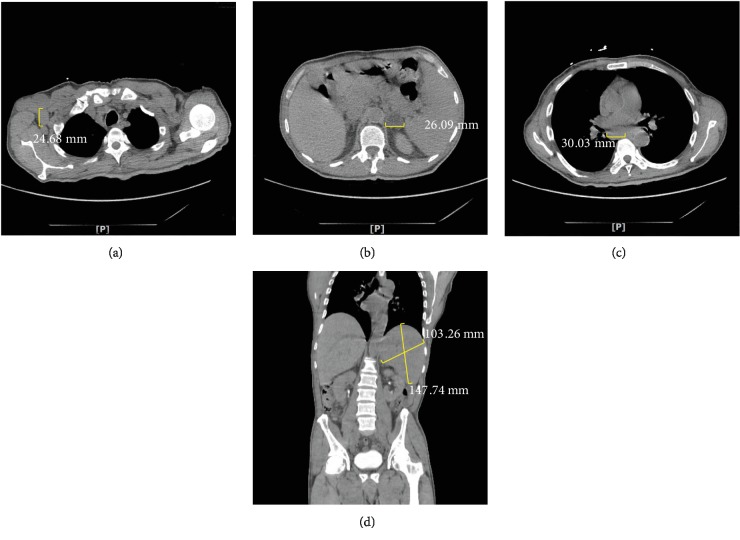
CT of the chest, abdomen, and pelvis. (a) Axillary lymphadenopathy. (b) Retroperitoneal lymphadenopathy. (c) Paraaortic lymphadenopathy. (d) Splenomegaly.

**Figure 3:**
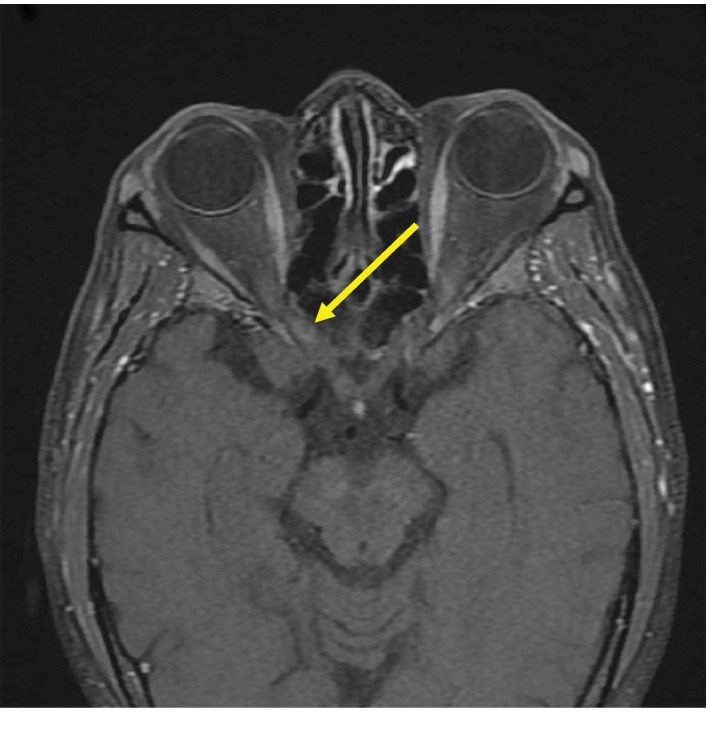
MRI of the brain and orbits with contrast showing lymphoma infiltration in the right optic nerve.

Lab work was significant for leukocytosis of 47,600/*μ*L with 86% lymphocytes (10% atypical lymphocytes), hemoglobin of 8.3 g/dL, platelet count of 46,000/*μ*L, and LDH of 4067 U/L. Due to the high leukocytosis and acute vision loss, there was concern for a retinopathy secondary to hyperviscosity syndrome. However, serum viscosity was 1.1 cP (range: 1.1-1.8 cP). Furthermore, CTA of the head and neck and carotid ultrasound were unremarkable showing no significant stenoses, which ruled out hyperviscosity syndrome and any vascular etiology for his vision loss. MRI of the brain showed no evidence of acute infarction or any enhancing mass lesion, which ruled out any acute etiology within the brain as the cause of his vision loss. CT of the chest, abdominal, and pelvis showed extensive mediastinal and retroperitoneal lymphadenopathy with splenomegaly (Figure 2). MRI of the brain and orbits showed a minimal focal area of enhancement within the right optic nerve inside of the optic canal (Figure 3).

Peripheral blood smear showed normocytic, hypochromic red blood cells, lymphocytosis comprised of small and large lymphocytes with few atypical lymphocytes, and thrombocytopenia without clumping. Biopsy was obtained from a paraaortic lymph node instead of the enlarged cervical lymph nodes to differentiate prostate cancer metastasis from a primary malignancy such as leukemia or lymphoma, as prostate cancer is unlikely to metastasize in cervical lymph nodes. The biopsy was consistent with B-cell chronic lymphocytic leukemia. Flow cytometry was positive for CD38 and ZAP-70. Bone marrow biopsy showed chronic lymphocytic leukemia greater than 90% of the cellularity with near total displacement by small lymphocytic infiltrate. Fluorescence in situ hybridization was positive for trisomy 12 and deletion of TP53 on chromosome 17 at p13. Immunohistochemical analysis of a paraaortic lymph node was positive for CD20, PAX5, CD5, CD23, BCL2, and CD43, but negative for Cyclin D1 and p53.

The patient was diagnosed with extensive stage IV small cell lymphocytic lymphoma with infiltration of the right posterior optic nerve. The patient was started on a regimen of rituximab and bendamustine. However, there was a concern that chemotherapy would not adequately penetrate the blood brain barrier to decrease the size of the intracranial lesion. Thus, palliative local radiation was initiated. Through radiation treatments directed at the lesion on his optic nerve, the patient slowly regained vision in his right eye.

## 3. Discussion

Chronic lymphocytic leukemia (CLL) is caused by the dysregulated growth and differentiation of malignant white blood cells in the bone marrow. These malignant white blood cells present in the blood often infiltrate other organs, such as the liver, spleen, and skin. Small lymphocytic leukemia (SLL) is a variant of CLL, in which most of the malignant white blood cells are present in lymph nodes. A previous study done in 2016 showed that any significant central nervous system involvement of CLL/SLL was an extremely rare consequence, with about 80% of the CNS symptoms occurring due to other causes [2]. CNS involvement in CLL/SLL is often undiagnosed or does not show any clinical signs or symptoms, but patients with CNS involvement have a median survival time of 12 months after diagnosis [3]. Studies have shown that CNS involvement in the progression of CLL/SLL occur in untreated patients while correlation with stage, duration, gender, age, WBC count, or immunologic phenotype has not been shown [4].

While the mechanism of spread into the CNS is not well understood to this point, several cases have been presented that postulate possible sites of entry into the CNS. The dura can act as a barrier to prevent direct invasion into the deeper layers of the CNS. However, spread into the CNS may come as a result of hematogenous or lymphomatous spread that perforates dural vessels and nerves into the arachnoid space [5]. Some studies have linked integrin CD49d, the *α* chain of the *α*4*β*1, with migration of malignant cells across vascular endothelium, showing clinical relevance to disease progression. In addition, extensive bone marrow infiltration has been linked to high CD49d expression [6].

The differential diagnosis for this patient, who presented with sudden onset right eye vision loss, is broad. Important diagnoses to consider include optic neuropathy, optic neuritis, autoimmune disorders, inflammatory pseudotumors, and other compressive neuropathies, including optic nerve glioma, optic nerve meningioma, optic nerve or orbital metastases, inherited disorder, and toxic, nutritional, or metabolic optic neuropathies [7]. Urgent ophthalmology consult may be necessary in order to influence the differential, ordering of subsequent testing, and prevention of further progression of the disease. If there is high clinical suspicion of CLL/SLL involvement in the optic nerve, immediate orbital radiation and induction chemotherapy may be necessary to preserve vision. MRI of the brain and orbit should be done as soon as possible to narrow down the differential, visualize any possible compressive lesions, and determine location of the disease [8].

Optic nerve biopsy can also be considered; however, this carries the risk of permanent vision loss and results may vary due to small biopsy collection in order to preserve vision [9]. In a study performed by Levin et al., 14 of 15 patients that underwent optic nerve biopsy were able to get a definitive diagnosis. Of those patients, 4 who had an unaffected fellow eye were prevented from further vision loss, and 6 patients with tumors had the etiology of their vision loss confirmed [10]. Kim et al. presented two cases in which patients with B-cell non-Hodgkin lymphoma had optic nerve involvement and underwent optic nerve biopsy. Results of one biopsy showed mononuclear cells invading the fibrovascular pial septa surrounding bundles of necrotic fibers. The other showed the fibrovascular septa of the optic nerve infiltrated by lymphocytes and histiocytes [5]. Optic nerve biopsy results were also presented in a case by Myers et al. in a patient with recurrent vision loss and a history of T lymphoblastic lymphoma in remission. The biopsy showed lymphoblastic infiltration with atypical lymphoid cells with morphology like the original disease [8].

Standard treatment for early, stable CLL disease is observation monitoring. For initial management of CLL in advanced and active disease, ibrutinib (NCCN Category 1) or venetoclax plus obinutuzumab (NCCN Category 2A) are preferred treatment options regardless of del(17p)/TP53 mutation status. Other alternative first-line therapy options include chlorambucil plus anti-CD20 antibody (NCCN Category 2A; ESMO Grade A, Level 1) or bendamustine plus anti-CD20 monoclonal antibody (ESMO Grade B, Level 1). Our patient was placed on rituximab and bendamustine as it was thought to be better tolerated in an older patient than ibrutinib due to its toxicities with his multiple comorbidities and poor prognosis. For patients with SLL, regional radiation is often used. Lymphomas are among the group of tumors that are exquisitely sensitive to radiation therapy. In a study done by Johannsson et al., patients with disseminated CLL and indolent non-Hodgkin lymphoma (iNHL) were treated with low-dose radiotherapy (4 Gy in 2 fractions) for palliative treatment. Patients with CLL had an overall response rate of 71% (29% complete response, 42% partial response) with a median duration of response of about 22 months and no significant side effects. The study concluded that low-dose radiotherapy is a highly effective palliative treatment for masses in patients with disseminated CLL and iNHL [11]. In a study done by Pacelli et al., rare orbital tumors treated with radiotherapy had subjective and objective improvement of visual and ocular symptoms without evidence of disease progression [12].

Our case showed the rapid progression of the disease within the span of 6 months from the first clinically reported symptoms. The patient experienced severe neurologic symptoms, including complete right-sided, painless vision loss and right-sided headache and neck pain. The patient had a history of lymphadenopathy without a diagnosis of CLL/SLL, further complicating the case. While the clinical manifestations of this type of leukemia can be variable, CNS involvement provides a particularly unfavorable prognosis. However, the role of CNS involvement in SLL should be studied further to better understand disease progression.

## 4. Conclusion

SLL is an indolent form of lymphoma and patients often experience asymptomatic lymphadenopathy with no other symptoms. While cytopenias and bone marrow failure are common, neurologic manifestations of SLL are very rare but should be included in the differential of patients with sudden onset neurological symptoms and progressive lymphadenopathy.
